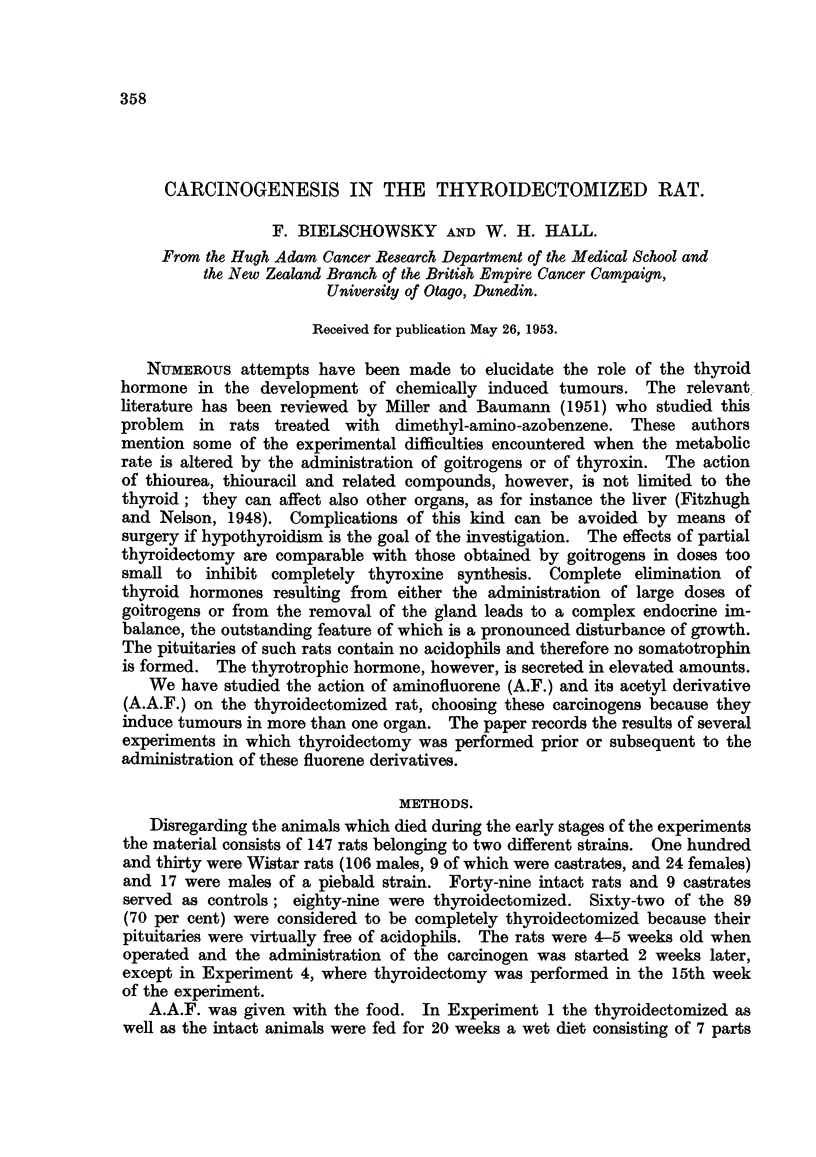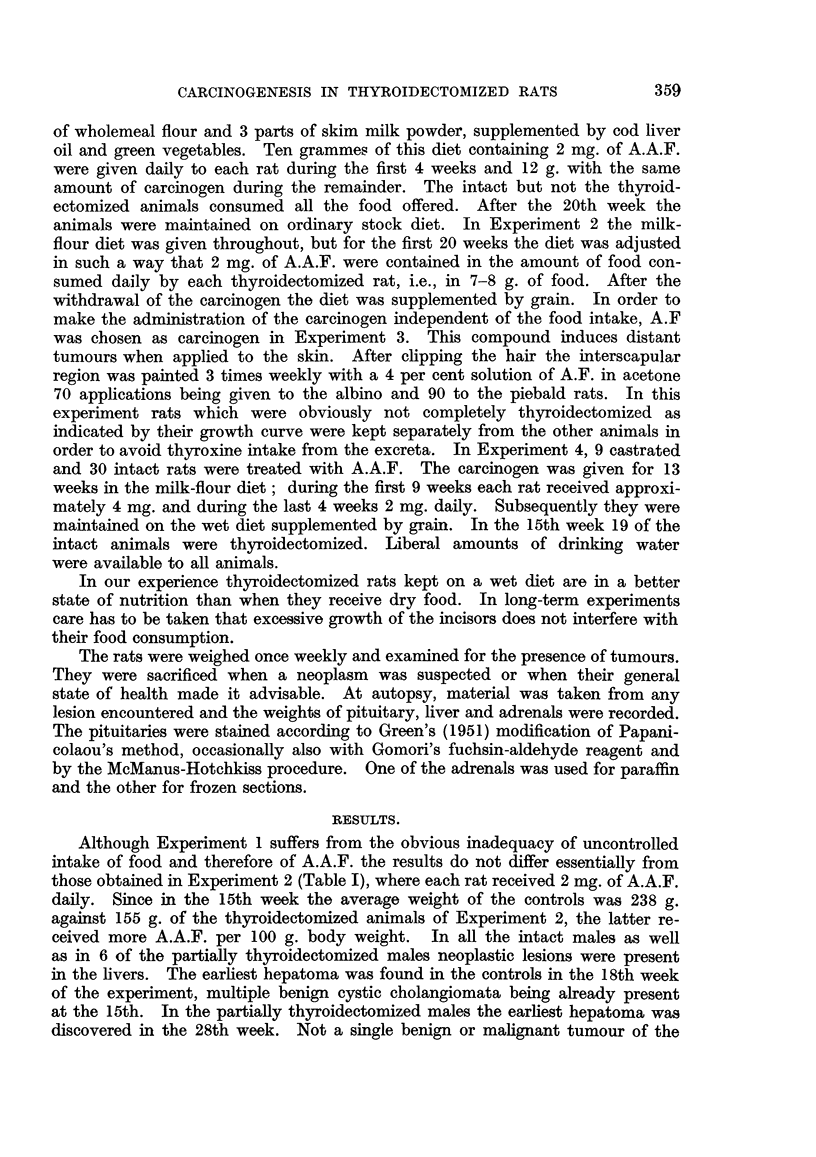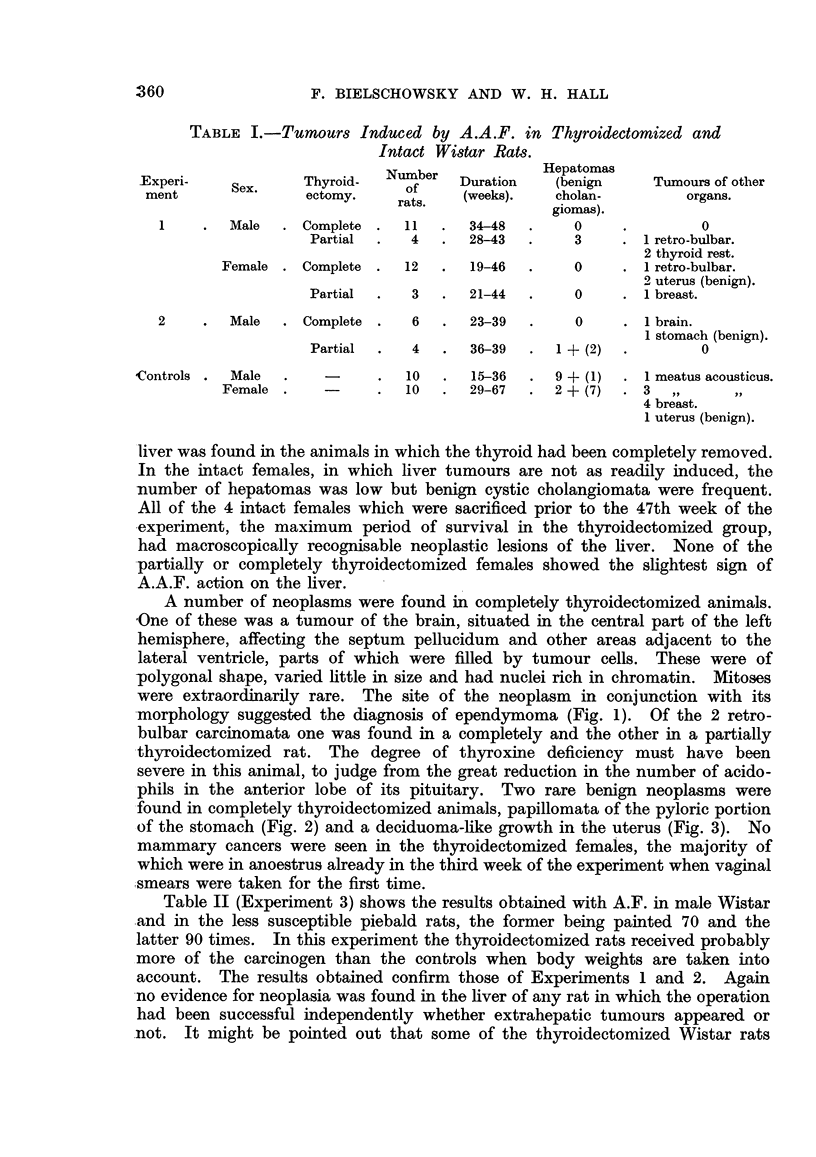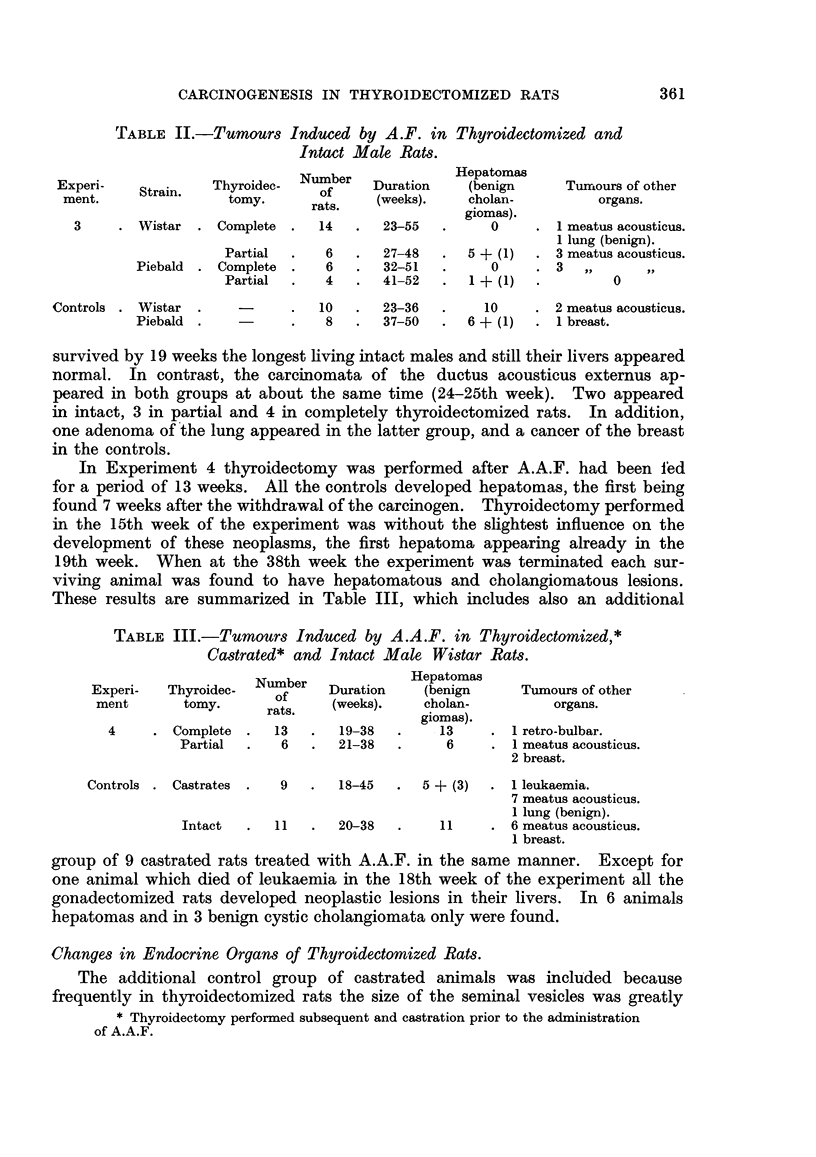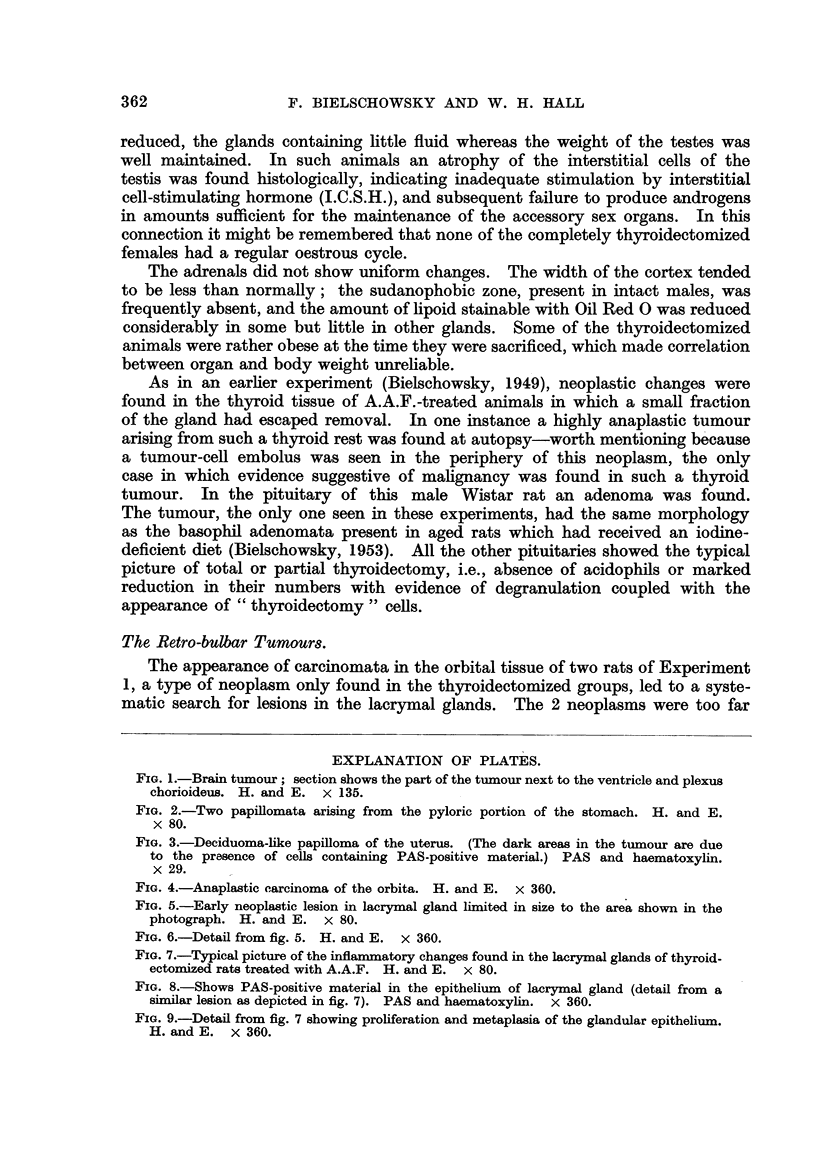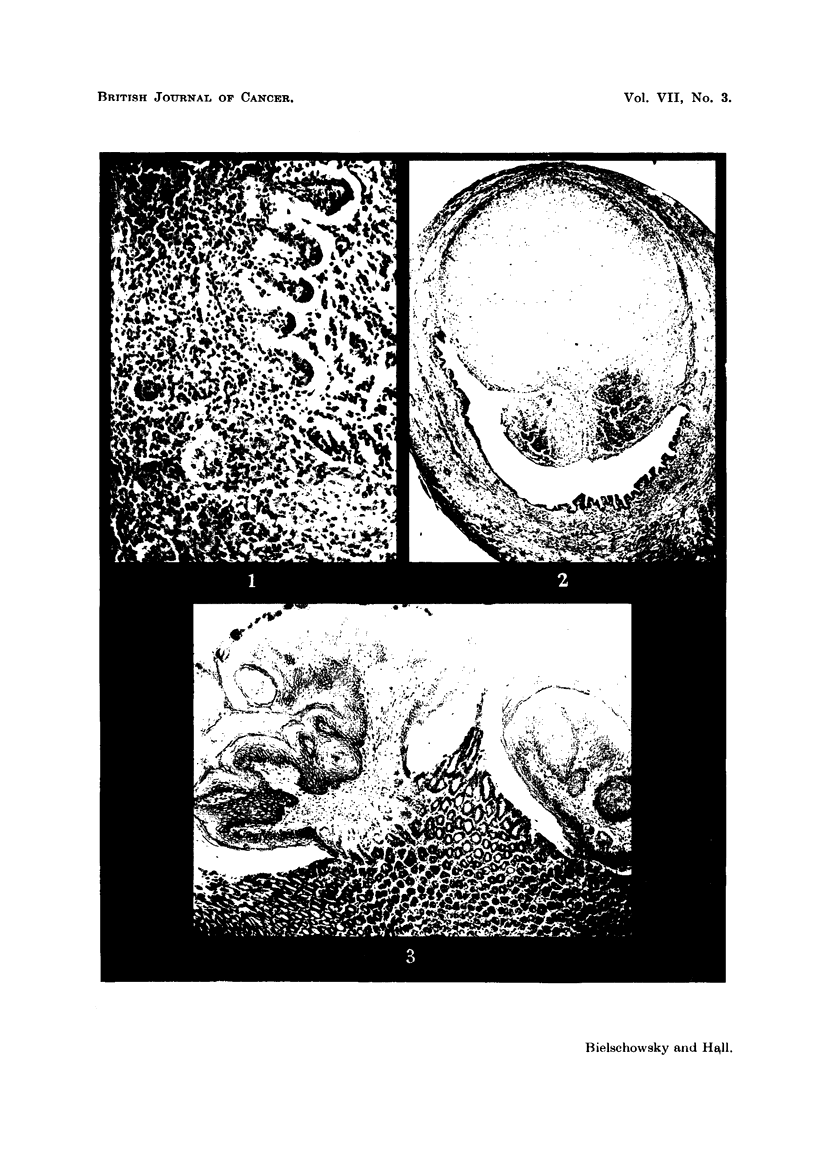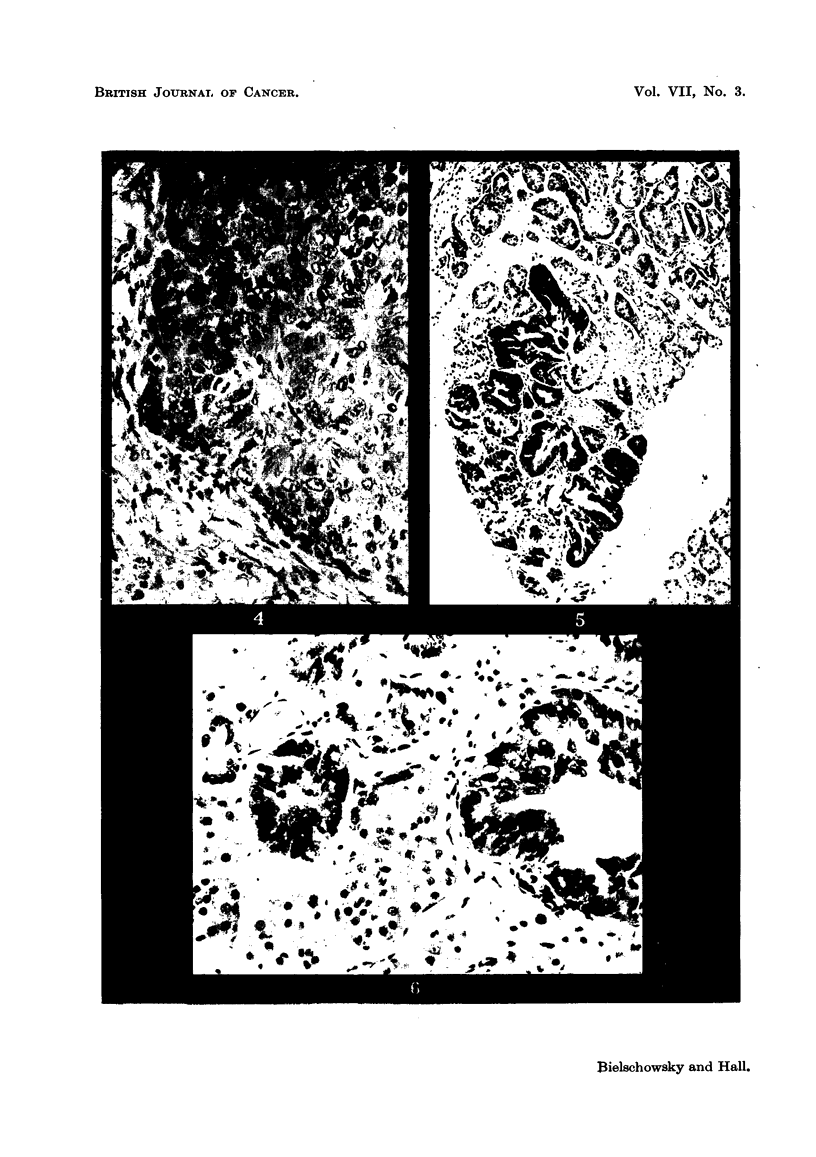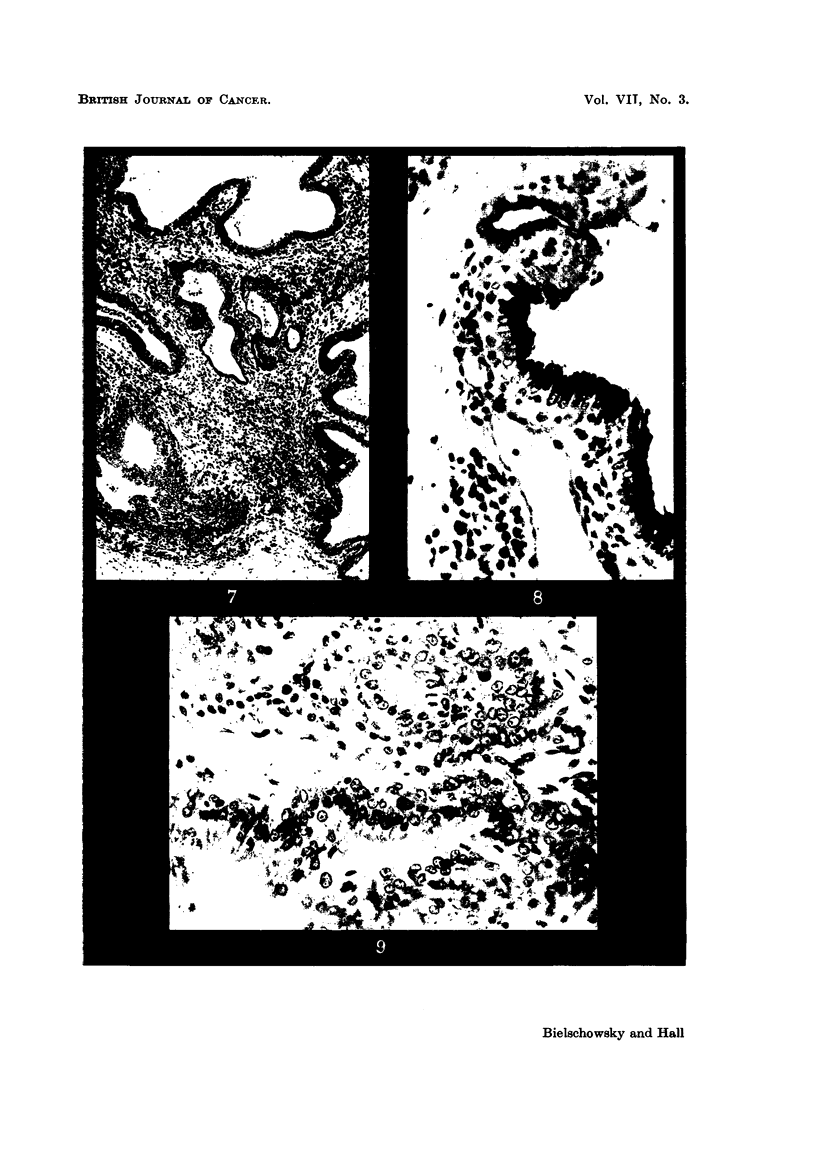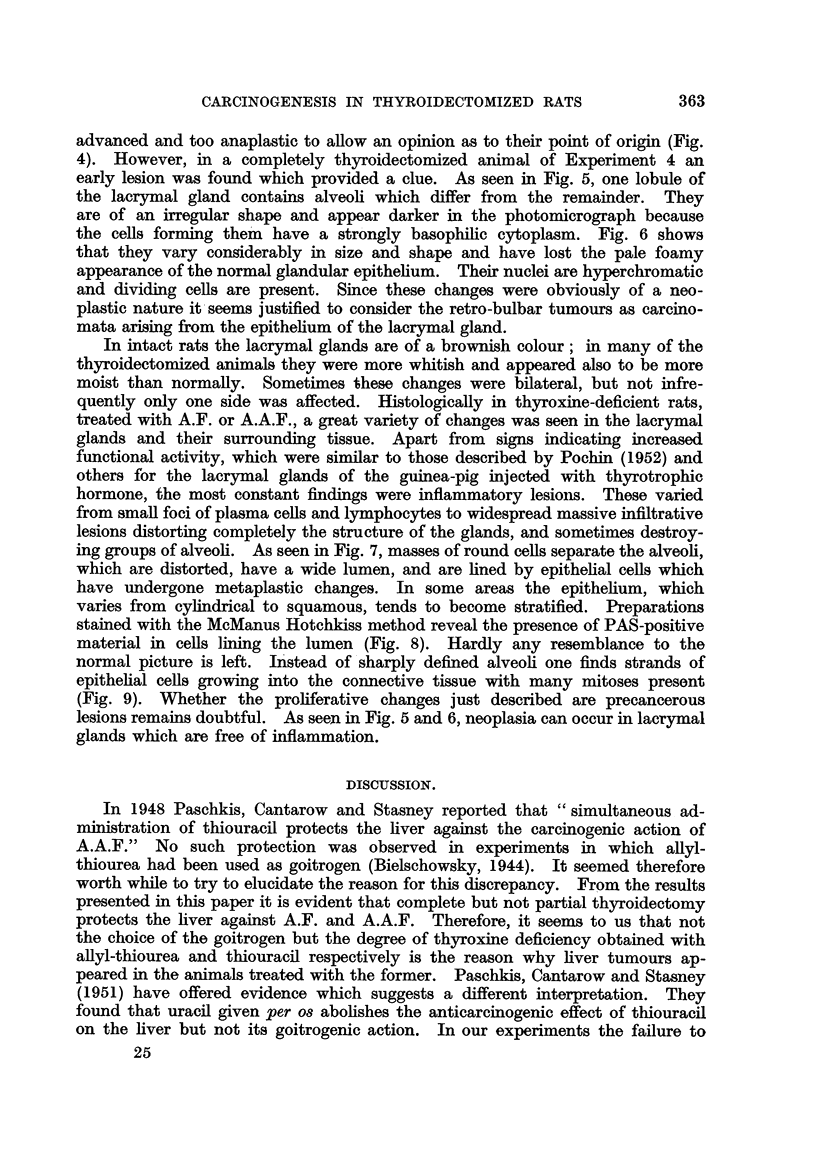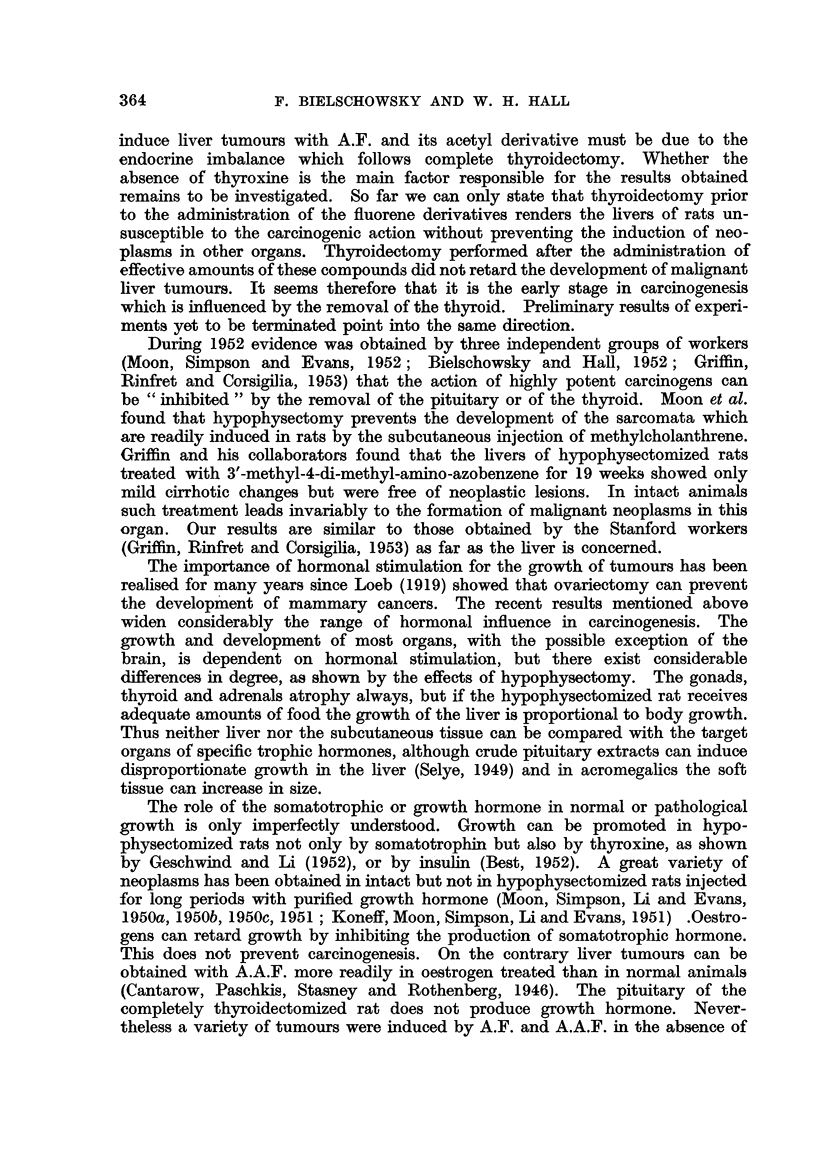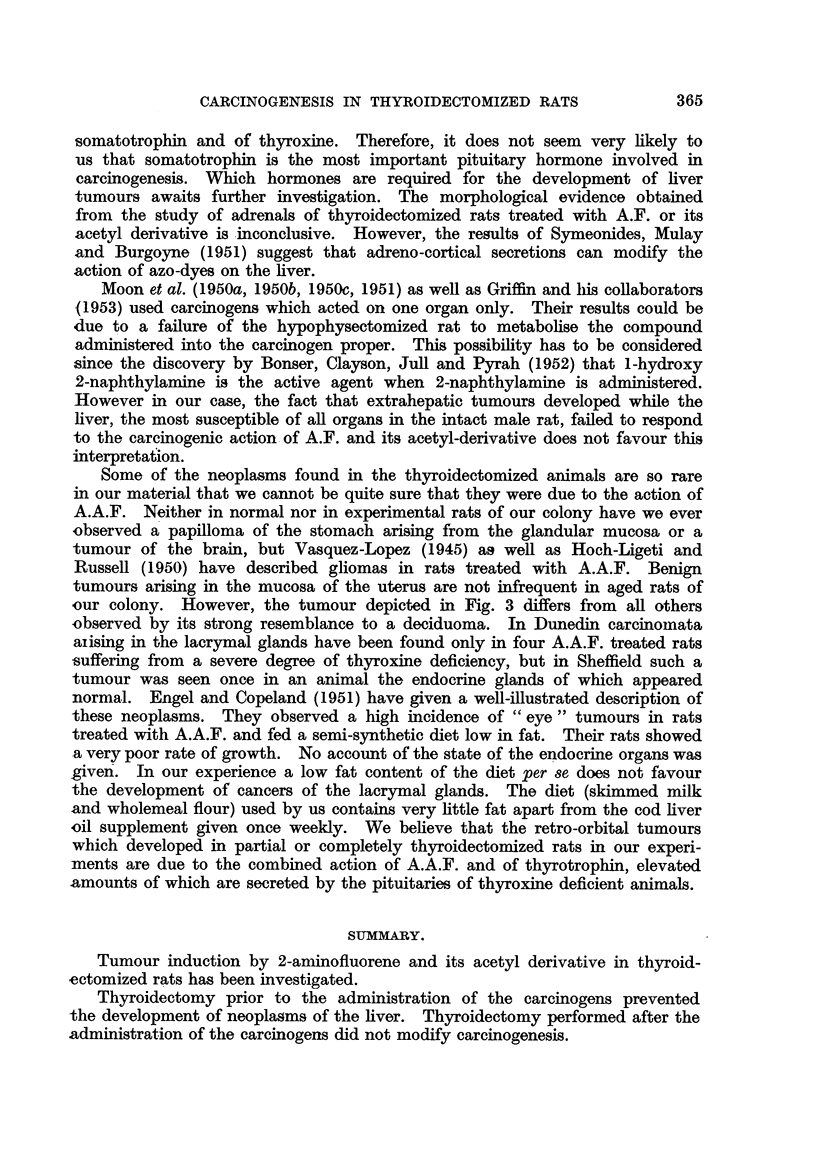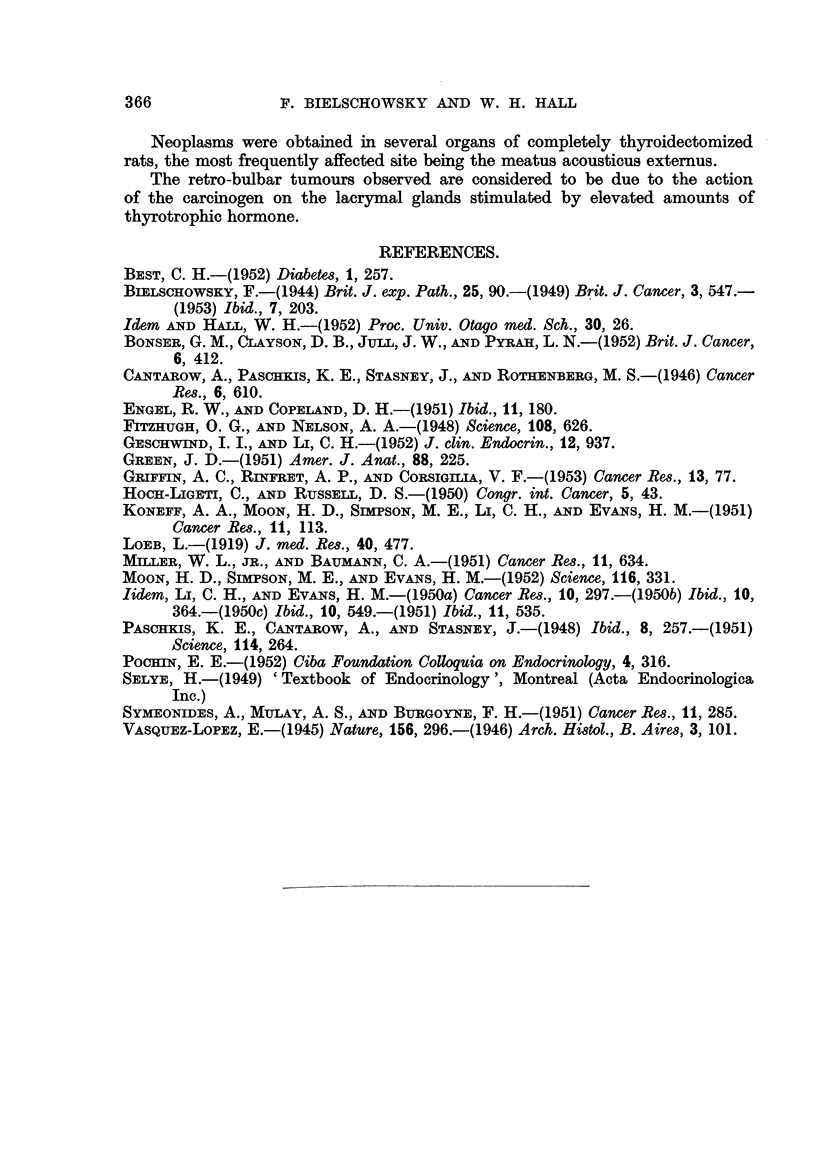# Carcinogenesis in the Thyroidectomized Rat

**DOI:** 10.1038/bjc.1953.34

**Published:** 1953-09

**Authors:** F. Bielschowsky, W. H. Hall

## Abstract

**Images:**


### CARCINOGENESIS IN THE THYROIDECTOMIZED RAT.

F. BIELSCHOWSKY AND W. H. HALL.

*From the Hugh Adam Cancer Research Department of the Medical School and the New Zealand Branch of the British Empire Cancer Campaign, University of Otago, Dunedin.*

Received for publication May 26, 1953.

NUMEROUS attempts have been made to elucidate the role of the thyroid hormone in the development of chemically induced tumours. The relevant literature has been reviewed by Miller and Baumann (1951) who studied this problem in rats treated with dimethyl-amino-azobenzene. These authors mention some of the experimental difficulties encountered when the metabolic rate is altered by the administration of goitrogens or of thyroxin. The action of thiourea, thiouracil and related compounds, however, is not limited to the thyroid ; they can affect also other organs, as for instance the liver (Fitzhugh and Nelson, 1948). Complications of this kind can be avoided by means of surgery if hypothyroidism is the goal of the investigation. The effects of partial thyroidectomy are comparable with those obtained by goitrogens in doses too small to inhibit completely thyroxine synthesis. Complete elimination of thyroid hormones resulting from either the administration of large doses of goitrogens or from the removal of the gland leads to a complex endocrine imbalance, the outstanding feature of which is a pronounced disturbance of growth. The pituitaries of such rats contain no acidophils and therefore no somatotrophin is formed. The thyrotrophic hormone, however, is secreted in elevated amounts.

We have studied the action of aminofluorene (A.F.) and its acetyl derivative (A.A.F.) on the thyroidectomized rat, choosing these carcinogens because they induce tumours in more than one organ. The paper records the results of several experiments in which thyroidectomy was performed prior or subsequent to the administration of these fluorene derivatives.

### METHODS.

Disregarding the animals which died during the early stages of the experiments the material consists of 147 rats belonging to two different strains. One hundred and thirty were Wistar rats (106 males, 9 of which were castrates, and 24 females) and 17 were males of a piebald strain. Forty-nine intact rats and 9 castrates served as controls ; eighty-nine were thyroidectomized. Sixty-two of the 89 (70 per cent) were considered to be completely thyroidectomized because their pituitaries were virtually free of acidophils. The rats were 4–5 weeks old when operated and the administration of the carcinogen was started 2 weeks later, except in Experiment 4, where thyroidectomy was performed in the 15th week of the experiment.

A.A.F. was given with the food. In Experiment 1 the thyroidectomized as well as the intact animals were fed for 20 weeks a wet diet consisting of 7 parts of wholemeal flour and 3 parts of skim milk powder, supplemented by cod liver oil and green vegetables. Ten grammes of this diet containing 2 mg. of A.A.F. were given daily to each rat during the first 4 weeks and 12 g. with the same amount of carcinogen during the remainder. The intact but not the thyroidectomized animals consumed all the food offered. After the 20th week the animals were maintained on ordinary stock diet. In Experiment 2 the milk-flour diet was given throughout, but for the first 20 weeks the diet was adjusted in such a way that 2 mg. of A.A.F. were contained in the amount of food consumed daily by each thyroidectomized rat, i.e., in 7–8 g. of food. After the withdrawal of the carcinogen the diet was supplemented by grain. In order to make the administration of the carcinogen independent of the food intake, A.F was chosen as carcinogen in Experiment 3. This compound induces distant tumours when applied to the skin. After clipping the hair the interscapular region was painted 3 times weekly with a 4 per cent solution of A.F. in acetone 70 applications being given to the albino and 90 to the piebald rats. In this experiment rats which were obviously not completely thyroidectomized as indicated by their growth curve were kept separately from the other animals in order to avoid thyroxine intake from the excreta. In Experiment 4, 9 castrated and 30 intact rats were treated with A.A.F. The carcinogen was given for 13 weeks in the milk-flour diet ; during the first 9 weeks each rat received approximately 4 mg. and during the last 4 weeks 2 mg. daily. Subsequently they were maintained on the wet diet supplemented by grain. In the 15th week 19 of the intact animals were thyroidectomized. Liberal amounts of drinking water were available to all animals.

In our experience thyroidectomized rats kept on a wet diet are in a better state of nutrition than when they receive dry food. In long-term experiments care has to be taken that excessive growth of the incisors does not interfere with their food consumption.

The rats were weighed once weekly and examined for the presence of tumours. They were sacrificed when a neoplasm was suspected or when their general state of health made it advisable. At autopsy, material was taken from any lesion encountered and the weights of pituitary, liver and adrenals were recorded. The pituitaries were stained according to Green's (1951) modification of Papanicolaou's method, occasionally also with Gomori's fuchsin-aldehyde reagent and by the McManus-Hotchkiss procedure. One of the adrenals was used for paraffin and the other for frozen sections.

### RESULTS.

Although Experiment 1 suffers from the obvious inadequacy of uncontrolled intake of food and therefore of A.A.F. the results do not differ essentially from those obtained in Experiment 2 (Table I), where each rat received 2 mg. of A.A.F. daily. Since in the 15th week the average weight of the controls was 238 g. against 155 g. of the thyroidectomized animals of Experiment 2, the latter received more A.A.F. per 100 g. body weight. In all the intact males as well as in 6 of the partially thyroidectomized males neoplastic lesions were present in the livers. The earliest hepatoma was found in the controls in the 18th week of the experiment, multiple benign cystic cholangiomata being already present at the 15th. In the partially thyroidectomized males the earliest hepatoma was discovered in the 28th week. Not a single benign or malignant tumour of the

TABLE I.—*Tumours Induced by A.A.F. in Thyroidectomized and Intact Wistar Rats.*

| Experiment | Sex. | Thyroidectomy. | Number of rats. | Duration (weeks). | Hepatomas (benign cholangiomas). | Tumours of other organs. |
|---|---|---|---|---|---|---|
| 1 | Male | Complete | 11 | 34–48 | 0 | 0 |
| | | Partial | 4 | 28–43 | 3 | 1 retro-bulbar. 2 thyroid rest. |
| | Female | Complete | 12 | 19–46 | 0 | 1 retro-bulbar. 2 uterus (benign). |
| | | Partial | 3 | 21–44 | 0 | 1 breast. |
| 2 | Male | Complete | 6 | 23–39 | 0 | 1 brain. 1 stomach (benign). |
| | | Partial | 4 | 36–39 | 1 + (2) | 0 |
| Controls | Male | — | 10 | 15–36 | 9 + (1) | 1 meatus acousticus. |
| | Female | — | 10 | 29–67 | 2 + (7) | 3 ,, ,, 4 breast. 1 uterus (benign). |

liver was found in the animals in which the thyroid had been completely removed. In the intact females, in which liver tumours are not as readily induced, the number of hepatomas was low but benign cystic cholangiomata were frequent. All of the 4 intact females which were sacrificed prior to the 47th week of the experiment, the maximum period of survival in the thyroidectomized group, had macroscopically recognisable neoplastic lesions of the liver. None of the partially or completely thyroidectomized females showed the slightest sign of A.A.F. action on the liver.

A number of neoplasms were found in completely thyroidectomized animals. One of these was a tumour of the brain, situated in the central part of the left hemisphere, affecting the septum pellucidum and other areas adjacent to the lateral ventricle, parts of which were filled by tumour cells. These were of polygonal shape, varied little in size and had nuclei rich in chromatin. Mitoses were extraordinarily rare. The site of the neoplasm in conjunction with its morphology suggested the diagnosis of ependymoma (Fig. 1). Of the 2 retrobulbar carcinomata one was found in a completely and the other in a partially thyroidectomized rat. The degree of thyroxine deficiency must have been severe in this animal, to judge from the great reduction in the number of acidophils in the anterior lobe of its pituitary. Two rare benign neoplasms were found in completely thyroidectomized animals, papillomata of the pyloric portion of the stomach (Fig. 2) and a deciduoma-like growth in the uterus (Fig. 3). No mammary cancers were seen in the thyroidectomized females, the majority of which were in anoestrus already in the third week of the experiment when vaginal smears were taken for the first time.

Table II (Experiment 3) shows the results obtained with A.F. in male Wistar and in the less susceptible piebald rats, the former being painted 70 and the latter 90 times. In this experiment the thyroidectomized rats received probably more of the carcinogen than the controls when body weights are taken into account. The results obtained confirm those of Experiments 1 and 2. Again no evidence for neoplasia was found in the liver of any rat in which the operation had been successful independently whether extrahepatic tumours appeared or not. It might be pointed out that some of the thyroidectomized Wistar rats

TABLE II.—*Tumours Induced by A.F. in Thyroidectomized and Intact Male Rats.*

| Experiment. | Strain. | Thyroidectomy. | Number of rats. | Duration (weeks). | Hepatomas (benign cholangiomas). | Tumours of other organs. |
|---|---|---|---|---|---|---|
| 3 | Wistar | Complete | 14 | 23–55 | 0 | 1 meatus acousticus.<br>1 lung (benign). |
| | | Partial | 6 | 27–48 | 5 + (1) | 3 meatus acousticus. |
| | Piebald | Complete | 6 | 32–51 | 0 | 3 " " |
| | | Partial | 4 | 41–52 | 1 + (1) | 0 |
| Controls | Wistar | — | 10 | 23–36 | 10 | 2 meatus acousticus. |
| | Piebald | — | 8 | 37–50 | 6 + (1) | 1 breast. |

survived by 19 weeks the longest living intact males and still their livers appeared normal. In contrast, the carcinomata of the ductus acousticus externus appeared in both groups at about the same time (24–25th week). Two appeared in intact, 3 in partial and 4 in completely thyroidectomized rats. In addition, one adenoma of the lung appeared in the latter group, and a cancer of the breast in the controls.

In Experiment 4 thyroidectomy was performed after A.A.F. had been fed for a period of 13 weeks. All the controls developed hepatomas, the first being found 7 weeks after the withdrawal of the carcinogen. Thyroidectomy performed in the 15th week of the experiment was without the slightest influence on the development of these neoplasms, the first hepatoma appearing already in the 19th week. When at the 38th week the experiment was terminated each surviving animal was found to have hepatomatous and cholangiomatous lesions. These results are summarized in Table III, which includes also an additional

TABLE III.—*Tumours Induced by A.A.F. in Thyroidectomized,* Castrated* and Intact Male Wistar Rats.*

| Experiment | Thyroidectomy. | Number of rats. | Duration (weeks). | Hepatomas (benign cholangiomas). | Tumours of other organs. |
|---|---|---|---|---|---|
| 4 | Complete | 13 | 19–38 | 13 | 1 retro-bulbar. |
| | Partial | 6 | 21–38 | 6 | 1 meatus acousticus.<br>2 breast. |
| Controls | Castrates | 9 | 18–45 | 5 + (3) | 1 leukaemia.<br>7 meatus acousticus.<br>1 lung (benign). |
| | Intact | 11 | 20–38 | 11 | 6 meatus acousticus.<br>1 breast. |

group of 9 castrated rats treated with A.A.F. in the same manner. Except for one animal which died of leukaemia in the 18th week of the experiment all the gonadectomized rats developed neoplastic lesions in their livers. In 6 animals hepatomas and in 3 benign cystic cholangiomata only were found.

*Changes in Endocrine Organs of Thyroidectomized Rats.*

The additional control group of castrated animals was included because frequently in thyroidectomized rats the size of the seminal vesicles was greatly

* Thyroidectomy performed subsequent and castration prior to the administration of A.A.F.

reduced, the glands containing little fluid whereas the weight of the testes was well maintained. In such animals an atrophy of the interstitial cells of the testis was found histologically, indicating inadequate stimulation by interstitial cell-stimulating hormone (I.C.S.H.), and subsequent failure to produce androgens in amounts sufficient for the maintenance of the accessory sex organs. In this connection it might be remembered that none of the completely thyroidectomized females had a regular oestrous cycle.

The adrenals did not show uniform changes. The width of the cortex tended to be less than normally ; the sudanophobic zone, present in intact males, was frequently absent, and the amount of lipoid stainable with Oil Red O was reduced considerably in some but little in other glands. Some of the thyroidectomized animals were rather obese at the time they were sacrificed, which made correlation between organ and body weight unreliable.

As in an earlier experiment (Bielschowsky, 1949), neoplastic changes were found in the thyroid tissue of A.A.F.-treated animals in which a small fraction of the gland had escaped removal. In one instance a highly anaplastic tumour arising from such a thyroid rest was found at autopsy—worth mentioning because a tumour-cell embolus was seen in the periphery of this neoplasm, the only case in which evidence suggestive of malignancy was found in such a thyroid tumour. In the pituitary of this male Wistar rat an adenoma was found. The tumour, the only one seen in these experiments, had the same morphology as the basophil adenomata present in aged rats which had received an iodine-deficient diet (Bielschowsky, 1953). All the other pituitaries showed the typical picture of total or partial thyroidectomy, i.e., absence of acidophils or marked reduction in their numbers with evidence of degranulation coupled with the appearance of " thyroidectomy " cells.

*The Retro-bulbar Tumours.*

The appearance of carcinomata in the orbital tissue of two rats of Experiment 1, a type of neoplasm only found in the thyroidectomized groups, led to a systematic search for lesions in the lacrymal glands. The 2 neoplasms were too far

---

EXPLANATION OF PLATES.

FIG. 1.—Brain tumour ; section shows the part of the tumour next to the ventricle and plexus chorioideus. H. and E. × 135.

FIG. 2.—Two papillomata arising from the pyloric portion of the stomach. H. and E. × 80.

FIG. 3.—Deciduoma-like papilloma of the uterus. (The dark areas in the tumour are due to the presence of cells containing PAS-positive material.) PAS and haematoxylin. × 29.

FIG. 4.—Anaplastic carcinoma of the orbita. H. and E. × 360.

FIG. 5.—Early neoplastic lesion in lacrymal gland limited in size to the area shown in the photograph. H. and E. × 80.

FIG. 6.—Detail from fig. 5. H. and E. × 360.

FIG. 7.—Typical picture of the inflammatory changes found in the lacrymal glands of thyroidectomized rats treated with A.A.F. H. and E. × 80.

FIG. 8.—Shows PAS-positive material in the epithelium of lacrymal gland (detail from a similar lesion as depicted in fig. 7). PAS and haematoxylin. × 360.

FIG. 9.—Detail from fig. 7 showing proliferation and metaplasia of the glandular epithelium. H. and E. × 360.

1

2

3

4

5

6

Bielschowsky and Hall.

7

8

9

advanced and too anaplastic to allow an opinion as to their point of origin (Fig. 4). However, in a completely thyroidectomized animal of Experiment 4 an early lesion was found which provided a clue. As seen in Fig. 5, one lobule of the lacrymal gland contains alveoli which differ from the remainder. They are of an irregular shape and appear darker in the photomicrograph because the cells forming them have a strongly basophilic cytoplasm. Fig. 6 shows that they vary considerably in size and shape and have lost the pale foamy appearance of the normal glandular epithelium. Their nuclei are hyperchromatic and dividing cells are present. Since these changes were obviously of a neoplastic nature it seems justified to consider the retro-bulbar tumours as carcinomata arising from the epithelium of the lacrymal gland.

In intact rats the lacrymal glands are of a brownish colour ; in many of the thyroidectomized animals they were more whitish and appeared also to be more moist than normally. Sometimes these changes were bilateral, but not infrequently only one side was affected. Histologically in thyroxine-deficient rats, treated with A.F. or A.A.F., a great variety of changes was seen in the lacrymal glands and their surrounding tissue. Apart from signs indicating increased functional activity, which were similar to those described by Pochin (1952) and others for the lacrymal glands of the guinea-pig injected with thyrotrophic hormone, the most constant findings were inflammatory lesions. These varied from small foci of plasma cells and lymphocytes to widespread massive infiltrative lesions distorting completely the structure of the glands, and sometimes destroying groups of alveoli. As seen in Fig. 7, masses of round cells separate the alveoli, which are distorted, have a wide lumen, and are lined by epithelial cells which have undergone metaplastic changes. In some areas the epithelium, which varies from cylindrical to squamous, tends to become stratified. Preparations stained with the McManus Hotchkiss method reveal the presence of PAS-positive material in cells lining the lumen (Fig. 8). Hardly any resemblance to the normal picture is left. Instead of sharply defined alveoli one finds strands of epithelial cells growing into the connective tissue with many mitoses present (Fig. 9). Whether the proliferative changes just described are precancerous lesions remains doubtful. As seen in Fig. 5 and 6, neoplasia can occur in lacrymal glands which are free of inflammation.

### DISCUSSION.

In 1948 Paschkis, Cantarow and Stasney reported that " simultaneous administration of thiouracil protects the liver against the carcinogenic action of A.A.F." No such protection was observed in experiments in which allyl-thiourea had been used as goitrogen (Bielschowsky, 1944). It seemed therefore worth while to try to elucidate the reason for this discrepancy. From the results presented in this paper it is evident that complete but not partial thyroidectomy protects the liver against A.F. and A.A.F. Therefore, it seems to us that not the choice of the goitrogen but the degree of thyroxine deficiency obtained with allyl-thiourea and thiouracil respectively is the reason why liver tumours appeared in the animals treated with the former. Paschkis, Cantarow and Stasney (1951) have offered evidence which suggests a different interpretation. They found that uracil given *per os* abolishes the anticarcinogenic effect of thiouracil on the liver but not its goitrogenic action. In our experiments the failure to induce liver tumours with A.F. and its acetyl derivative must be due to the endocrine imbalance which follows complete thyroidectomy. Whether the absence of thyroxine is the main factor responsible for the results obtained remains to be investigated. So far we can only state that thyroidectomy prior to the administration of the fluorene derivatives renders the livers of rats unsusceptible to the carcinogenic action without preventing the induction of neoplasms in other organs. Thyroidectomy performed after the administration of effective amounts of these compounds did not retard the development of malignant liver tumours. It seems therefore that it is the early stage in carcinogenesis which is influenced by the removal of the thyroid. Preliminary results of experiments yet to be terminated point into the same direction.

During 1952 evidence was obtained by three independent groups of workers (Moon, Simpson and Evans, 1952; Bielschowsky and Hall, 1952; Griffin, Rinfret and Corsigilia, 1953) that the action of highly potent carcinogens can be "inhibited" by the removal of the pituitary or of the thyroid. Moon *et al.* found that hypophysectomy prevents the development of the sarcomata which are readily induced in rats by the subcutaneous injection of methylcholanthrene. Griffin and his collaborators found that the livers of hypophysectomized rats treated with 3′-methyl-4-di-methyl-amino-azobenzene for 19 weeks showed only mild cirrhotic changes but were free of neoplastic lesions. In intact animals such treatment leads invariably to the formation of malignant neoplasms in this organ. Our results are similar to those obtained by the Stanford workers (Griffin, Rinfret and Corsigilia, 1953) as far as the liver is concerned.

The importance of hormonal stimulation for the growth of tumours has been realised for many years since Loeb (1919) showed that ovariectomy can prevent the development of mammary cancers. The recent results mentioned above widen considerably the range of hormonal influence in carcinogenesis. The growth and development of most organs, with the possible exception of the brain, is dependent on hormonal stimulation, but there exist considerable differences in degree, as shown by the effects of hypophysectomy. The gonads, thyroid and adrenals atrophy always, but if the hypophysectomized rat receives adequate amounts of food the growth of the liver is proportional to body growth. Thus neither liver nor the subcutaneous tissue can be compared with the target organs of specific trophic hormones, although crude pituitary extracts can induce disproportionate growth in the liver (Selye, 1949) and in acromegalics the soft tissue can increase in size.

The role of the somatotrophic or growth hormone in normal or pathological growth is only imperfectly understood. Growth can be promoted in hypophysectomized rats not only by somatotrophin but also by thyroxine, as shown by Geschwind and Li (1952), or by insulin (Best, 1952). A great variety of neoplasms has been obtained in intact but not in hypophysectomized rats injected for long periods with purified growth hormone (Moon, Simpson, Li and Evans, 1950*a*, 1950*b*, 1950*c*, 1951; Koneff, Moon, Simpson, Li and Evans, 1951) .Oestrogens can retard growth by inhibiting the production of somatotrophic hormone. This does not prevent carcinogenesis. On the contrary liver tumours can be obtained with A.A.F. more readily in oestrogen treated than in normal animals (Cantarow, Paschkis, Stasney and Rothenberg, 1946). The pituitary of the completely thyroidectomized rat does not produce growth hormone. Nevertheless a variety of tumours were induced by A.F. and A.A.F. in the absence of somatotrophin and of thyroxine. Therefore, it does not seem very likely to us that somatotrophin is the most important pituitary hormone involved in carcinogenesis. Which hormones are required for the development of liver tumours awaits further investigation. The morphological evidence obtained from the study of adrenals of thyroidectomized rats treated with A.F. or its acetyl derivative is inconclusive. However, the results of Symeonides, Mulay and Burgoyne (1951) suggest that adreno-cortical secretions can modify the action of azo-dyes on the liver.

Moon *et al.* (1950*a*, 1950*b*, 1950*c*, 1951) as well as Griffin and his collaborators (1953) used carcinogens which acted on one organ only. Their results could be due to a failure of the hypophysectomized rat to metabolise the compound administered into the carcinogen proper. This possibility has to be considered since the discovery by Bonser, Clayson, Jull and Pyrah (1952) that 1-hydroxy 2-naphthylamine is the active agent when 2-naphthylamine is administered. However in our case, the fact that extrahepatic tumours developed while the liver, the most susceptible of all organs in the intact male rat, failed to respond to the carcinogenic action of A.F. and its acetyl-derivative does not favour this interpretation.

Some of the neoplasms found in the thyroidectomized animals are so rare in our material that we cannot be quite sure that they were due to the action of A.A.F. Neither in normal nor in experimental rats of our colony have we ever observed a papilloma of the stomach arising from the glandular mucosa or a tumour of the brain, but Vasquez-Lopez (1945) as well as Hoch-Ligeti and Russell (1950) have described gliomas in rats treated with A.A.F. Benign tumours arising in the mucosa of the uterus are not infrequent in aged rats of our colony. However, the tumour depicted in Fig. 3 differs from all others observed by its strong resemblance to a deciduoma. In Dunedin carcinomata arising in the lacrymal glands have been found only in four A.A.F. treated rats suffering from a severe degree of thyroxine deficiency, but in Sheffield such a tumour was seen once in an animal the endocrine glands of which appeared normal. Engel and Copeland (1951) have given a well-illustrated description of these neoplasms. They observed a high incidence of " eye " tumours in rats treated with A.A.F. and fed a semi-synthetic diet low in fat. Their rats showed a very poor rate of growth. No account of the state of the endocrine organs was given. In our experience a low fat content of the diet *per se* does not favour the development of cancers of the lacrymal glands. The diet (skimmed milk and wholemeal flour) used by us contains very little fat apart from the cod liver oil supplement given once weekly. We believe that the retro-orbital tumours which developed in partial or completely thyroidectomized rats in our experiments are due to the combined action of A.A.F. and of thyrotrophin, elevated amounts of which are secreted by the pituitaries of thyroxine deficient animals.

### SUMMARY.

Tumour induction by 2-aminofluorene and its acetyl derivative in thyroidectomized rats has been investigated.

Thyroidectomy prior to the administration of the carcinogens prevented the development of neoplasms of the liver. Thyroidectomy performed after the administration of the carcinogens did not modify carcinogenesis.

Neoplasms were obtained in several organs of completely thyroidectomized rats, the most frequently affected site being the meatus acousticus externus.

The retro-bulbar tumours observed are considered to be due to the action of the carcinogen on the lacrymal glands stimulated by elevated amounts of thyrotrophic hormone.

### REFERENCES.

BEST, C. H.—(1952) *Diabetes*, **1**, 257.
BIELSCHOWSKY, F.—(1944) *Brit. J. exp. Path.*, **25**, 90.—(1949) *Brit. J. Cancer*, **3**, 547.—(1953) *Ibid.*, **7**, 203.
*Idem* AND HALL, W. H.—(1952) *Proc. Univ. Otago med. Sch.*, **30**, 26.
BONSER, G. M., CLAYSON, D. B., JULL, J. W., AND PYRAH, L. N.—(1952) *Brit. J. Cancer*, **6**, 412.
CANTAROW, A., PASCHKIS, K. E., STASNEY, J., AND ROTHENBERG, M. S.—(1946) *Cancer Res.*, **6**, 610.
ENGEL, R. W., AND COPELAND, D. H.—(1951) *Ibid.*, **11**, 180.
FITZHUGH, O. G., AND NELSON, A. A.—(1948) *Science*, **108**, 626.
GESCHWIND, I. I., AND LI, C. H.—(1952) *J. clin. Endocrin.*, **12**, 937.
GREEN, J. D.—(1951) *Amer. J. Anat.*, **88**, 225.
GRIFFIN, A. C., RINFRET, A. P., AND CORSIGILIA, V. F.—(1953) *Cancer Res.*, **13**, 77.
HOCH-LIGETI, C., AND RUSSELL, D. S.—(1950) *Congr. int. Cancer*, **5**, 43.
KONEFF, A. A., MOON, H. D., SIMPSON, M. E., LI, C. H., AND EVANS, H. M.—(1951) *Cancer Res.*, **11**, 113.
LOEB, L.—(1919) *J. med. Res.*, **40**, 477.
MILLER, W. L., JR., AND BAUMANN, C. A.—(1951) *Cancer Res.*, **11**, 634.
MOON, H. D., SIMPSON, M. E., AND EVANS, H. M.—(1952) *Science*, **116**, 331.
*Iidem*, LI, C. H., AND EVANS, H. M.—(1950*a*) *Cancer Res.*, **10**, 297.—(1950*b*) *Ibid.*, **10**, 364.—(1950*c*) *Ibid.*, **10**, 549.—(1951) *Ibid.*, **11**, 535.
PASCHKIS, K. E., CANTAROW, A., AND STASNEY, J.—(1948) *Ibid.*, **8**, 257.—(1951) *Science*, **114**, 264.
POCHIN, E. E.—(1952) *Ciba Foundation Colloquia on Endocrinology*, **4**, 316.
SELYE, H.—(1949) 'Textbook of Endocrinology', Montreal (Acta Endocrinologica Inc.)
SYMEONIDES, A., MULAY, A. S., AND BURGOYNE, F. H.—(1951) *Cancer Res.*, **11**, 285.
VASQUEZ-LOPEZ, E.—(1945) *Nature*, **156**, 296.—(1946) *Arch. Histol., B. Aires*, **3**, 101.